# Calcineurin subunit B is involved in shell regeneration in *Haliotis diversicolor*

**DOI:** 10.7717/peerj.10662

**Published:** 2021-01-12

**Authors:** Tiranan Buddawong, Somluk Asuvapongpatana, Chanyatip Suwannasing, Valainipha Habuddha, Chompoonut Sukonset, Chanyarak Sombutkayasith, Carmel McDougall, Wattana Weerachatyanukul

**Affiliations:** 1 Department of Anatomy, Faculty of Science, Mahidol University, Ratchathewi, Bangkok, Thailand; 2 Department of Radiological Technology, Faculty of Allied Health Sciences, Naresuan University, Mueang, Pitsanuloke, Thailand; 3 School of Allied Health Science, Walailak University, Thasala, Nakhon Si Thammarat, Thailand; 4 Australian Rivers Institute, Griffith University, Nathan, Queensland, Australia

**Keywords:** Shell, Regeneration, *Haliotis diversicolor*, Biomineralization, Calcineurin

## Abstract

Abalone shells are mainly composed of two major polymorphs of CaCO_3_ that are distributed in different layers of the shell. The process of shell biomineralization is controlled by genes and proteins expressed within the mantle epithelium. In this present paper, we conducted a shell regeneration experiment to study the role of HcCNA and HcCNB (individual subunits of calcineurin) in shell biomineralization in *H. diversicolor*. The results of qPCR showed that *HcCNB* is upregulated to a greater extent than *HcCNA* in the mantle after shell notching. In vivo study of the effects of rHcCNB injection showed a significantly higher percentage of regenerated shell length, but not area, in the injected group compared to the control group. In addition, SEM observation of the inner surface of the regenerated shells revealed three different zones including prismatic, nacreous, and a distinct transition zone. Changes in the crystal organization and ultrastructure are clearly evident in these three zones, particularly after 3 weeks of rHcCNB administration. We hypothesize that this is due to faster biomineralization rates in the rHcCNB treated group. Taken together, our results demonstrate that HcCNB participates in shell regeneration in *H. diversicolor*. As calcineurin subunits have also been implicated in shell formation in bivalves, these findings suggest that calcineurin subunits may play important roles in biomineralization in all conchiferans.

## Introduction

Calcineurin (CN), also called protein phosphatase 2B (PP2B), is the only member of the serine/threonine protein phosphatase family which can be activated by Ca^2+^ and calmodulin (a Ca^2+^-binding signaling protein) (Klee, Crouch & Krinks, 1979). Generally, CN is made up of two heterodimeric subunits, CNA and CNB. It has long been known that both subunits function synergistically; CNA acts as catalytic subunit of phosphatase while the basic function of CNB is to regulate the activity of CNA (Rusnak & Mertz, 2000). CNB is also reported to be able to function independently of CNA, by binding to proteasome subunit alpha type 7 which subsequently enhances proteasome pathway to cause an inhibition of hypoxia-inducible factor 1α (HIF-1α) activity (Li et al., 2011). CNB contains four EF-hand type Ca^2+^-binding motifs and can act as a Ca^2+^ chelator, and serum-based CNB can cause antiplatelet aggregation in rabbit blood (Su et al., 2011). The upregulation of CNB level over CNA has been reported in cancer tissues (Wei, Zhang & Wang, 1993), again suggesting an independent role for CNB, and the administration of recombinant human CNB showed a high anti-cancer potency with an unclear molecular mechanism (Jin et al., 2005). Recently, we also found that CNB has a more pronounced immuno-protective response than CNA in the abalone *Haliotis diversicolor* (a gastropod mollusk) in response to foreign microbial challenge (Buddawong et al., 2020). In this previous study we also noted constitutive expression of both *HcCNA* and *HcCNB* in the shell secreting epithelium, this, and the proposed role for calcineurin in shell formation in the bivalve *Pinctada fucata* (Li et al., 2010), prompted us to investigate the potential roles for calcineurin subunits in biomineralization in *H. diversicolor*.

In many mollusk shells, the outermost organic layer (called the periostracum) plays a vital role in sealing the extrapallial space, thus providing a primary template for mineralization (Marin, Roy & Marie, 2012). In abalone, subjacent to the periostracum is a mineralized layer defined as the prismatic layer which is made up of calcite or aragonite depending on the abalone species, followed by an innermost nacreous layer formed of aragonite tablets (Cusack et al., 2013). Shell biomineral products are composed of CaCO_3_ crystals, matrix protein, and other biopolymers (Wilbur, 1972; Mann, 2001). The secreted organic matrices are made up of numerous proteins; several of these have been well-characterized, and some are known to be the initiation site of mineral nucleation (Marin et al., 2000; Zhang et al., 2003; Marin & Luquet, 2005; Takeuchi & Endo, 2006; Zhang & Zhang, 2006). Together with matrix protein secretion, the following deposition of inorganic mineralization is under the control of a thin polarized epithelium, the mantle, which is known to synthesize and release the matrices for the shell mineralization process (Simkiss & Wilbur, 1989; Addadi et al., 2006). During shell damage in mollusks, the mantle epithelium adjacent to the inner rim becomes activated and promotes deposition of the mineralized crystals beneath the damaged shell (Fleury et al., 2008). After formation of the organic membranes, the crystallization of CaCO_3_ takes place through the amorphous phase followed by calcite and aragonite formation (Suzuki & Nagasawa, 2013; Hüning et al., 2016). Crystals of CaCO_3_ that formed in these layers vary in types and structures depending on the molluscan species and the region of the shell damage (Watabe, 1983). It should be noted that shell regeneration process may or may not be similar to the process of normal shell formation, particularly in their times and steps of molecular events taken place (Marin, Roy & Marie, 2012). In this study, we examined the responses of *HcCNA* and *HcCNB* genes during shell damage. The predominant effect of HcCNB (over HcCNA) was demonstrated by increased expression of *HcCNB* in the mantle following shell notching. Acceleration of shell regeneration during treatment with an exogenous recombinant HcCNB protein was also observed, providing evidence for its role in facilitating shell biomineralization.

## Materials and Methods

### Experimental animals, RNA isolation and cDNA synthesis

Adult abalone *H. diversicolor,* about 2 years of age, 55.0 mm in size and 10.0 g in weight, were reared in polyethylene tanks at 23−25 °C with a salinity of 28–30 ppt at Phuket Abalone Farm, Phuket, Thailand and fed daily with fresh kelp as previously described (Buddawong et al., 2020). Animal handling followed the guideline of Animal Care and Use Committee (SCMU-ACUC, Protocol Number MUSC60-040-390), Faculty of Science, Mahidol University. Prior to sampling, animals were anesthetized with 0.1 M KCl until no movement was detected. The mantle tissues were carefully dissected and stored in RNAlater RNA stabilization reagent (Ambion, Austin, TX, USA) prior to RNA extraction. Total RNA was extracted with Trizol reagent (Invitrogen, Carlsbad, CA, USA) according to the manufacturer’s protocol and genomic DNA was eliminated by DNAse treatment (Thermo Fisher Scientific, Carlsbad, CA, USA). RNA was reverse transcribed into cDNA using the SuperScript III First-Strand Synthesis System for RT-PCR (Invitrogen) with random hexamers following the manufacturer’s instructions.

### Shell notching experiment

To investigate the expression patterns of *HcCNA* and *HcCNB* in shell damaged abalone, the shell notching experiment was performed according to the method of (Mount et al., 2004) with some modifications. The V-shaped notch (approximately 10 mm^2^) was cut on the right margin of the abalone shells (*n* = 21) without damaging the mantle tissues. Animals were randomly divided into seven groups, each group containing three individuals. The seven groups were returned to seawater tanks and the abalones were sacrificed at 0, 3, 6, 12, 24, 36, and 48 h after shell notching. Approximately three cm^2^ area of the mantle tissue around the notching area was collected and immediately kept in RNAlater reagent for further extraction with Trizol reagent and DNAse treatment under the same condition described above. The mantle tissues of three abalones without shell notching were also collected as a control.

### Gene expression analysis by quantitative real-time PCR (qPCR)

qPCR was used to quantify the expression levels of *HcCNA* and *HcCNB*. As described previously (Buddawong et al., 2020), 1 µg of the total RNA from mantle was reverse transcribed into cDNA and qPCR was performed in triplicate using a Bio-Rad CFX96 Touch Real-Time PCR Detection System (Bio-Rad Laboratories, Inc., Hercules, CA, USA) with Luna Universal qPCR Master Mix (New England Biolabs, Ipswich, MA, USA). The 20 µl reaction mixture contained 0.5 µl (20 ng) of cDNA, 10 µl of Luna Universal qPCR mix with Hot Start *Taq* DNA Polymerase, 0.5 µl of 10 µM of each primer (for primer details see Table 1), and 8.5 µl of PCR grade water. The cycling parameters were 95 °C for 1 min, 45 cycles of 95 °C for 15 s, 60 °C for 30 s, and 72 °C for 30 s. A melting curve analysis was performed at a final single cycle to determine the specificity of PCR amplification by increasing the temperature from 60 °C to 95 °C in the rate of 0.05 °C/sec. The baseline was set automatically by Bio-Rad CFX manager software (version 3.1). PCRs with no template controls were also performed for each primer pair. Relative expression levels were calculated by the Livak (2^−ΔΔ*Cq*^) method using *β-actin* as a reference gene because of its previously determined stable expression in *H. diversicolor* (Li et al., 2012).

**Table 1:** Oligo nucleotide primers used in this study.

Primer name	Nucleotide sequence (5′→ 3′)	Purpose
*HcCNA*-F	AGGTGATCCGCAACAAAATC	qPCR
*HcCNA*-R	TCCTCCAGACAACACACCAA	
*HcCNB*-F	CAGTTTGCCAATGGAGCTTT	qPCR
*HcCNB*-R	CTCTCTGCACCAGTGGGTTT	
Expressed CNA-F	CCATGGGCCATCATCATCATCATCATGCTACGACCGATGGTAAG	Recombinant protein expression
Expressed CNA-R	GA GGATCCTTAGGAAGCCAGCTTCCTGCG	
Expressed CNB-F	GC CATATGGGAAATGAAAACAGTTTG	Recombinant protein expression
Expressed CNB-R	GA GGATCCTACGTCAACCACCATTTT	
*β-actin*-F	ACCACGGGTATTGTTCTTGAC	Reference gene
*β-actin*-R	CGGTGGTGGTGAAGGAGTAAC	

### Expression and purification of recombinant HcCNA (rHcCNA) and HcCNB (rHcCNB) in *E.coli*

To express the recombinant HcCNA and HcCNB in *E.coli*, the coding regions of HcCNA and HcCNB obtained from the previous study (Buddawong et al., 2020) were amplified by PCR using 2 pairs of primers (expressed CNA-F and expressed CNA-R for HcCNA and expressed CNB-F and expressed CNB-R for HcCNB) which contained the positions of NcoI/BamHI digestion and NdeI/BamHI digestion for HcCNA and HcCNB, respectively. The PCR products were purified with FavorPrep GEL/PCR purification kit (Favorgen, Ping-Tung, Taiwan) and digested with NcoI/BamHI and NdeI/BamHI for HcCNA and HcCNB, respectively. The digested HcCNA was inserted into pET-16b while that of digested HcCNB was inserted into pET-15b (Novagen, Darmstadt, Germany). The recombinant plasmids were confirmed by restriction analysis and DNA sequencing and transformed into *E. coli* strain BL21 (DE3, Novagen). Protein expression was induced with 1 mM isopropylthiogalactopyranoside (IPTG) at 37 °C when the optical density (600 nm) of the culture had reached 0.6. Bacterial cells were harvested by centrifugation (5,000× g, 10 min, 4 °C) after 4 h of induction.

For protein purification, the bacterial pellets were washed twice with PBS, centrifuged, and the bacterial pellets were suspended in ice-cold lysis buffer (50 mM Tris-HCl, 140 mM NaCl, 5 mM dithiothreitol (DTT), and 2 mM phenylmethylsulfonyl fluoride (PMSF), pH 8.0). Cell disruption was achieved by sonication on ice. After centrifugation (10,000× g, 30 min, 4 °C), crude proteins in supernatant were filtered through 0.45 µm sieve and loaded onto a nickel affinity chromatographic column. The column was washed with washing buffer (50 mM Tris-HCl, 140 mM NaCl, 20 mM imidazole, pH 8.0) and then eluted with elution buffer (50 mM Tris-HCl, 300 mM NaCl, 300 mM imidazole, pH 8.0).

### Protein profiling and Western blot analysis of rHcCNA and rHcCNB

The obtained fractions of rHcCNA and rHcCNB from the affinity column were analyzed by sodium dodecyl sulfate-polyacrylamine gel electrophoresis (SDS-PAGE) and stained with Coomassie Brilliant Blue. The dissolved proteins were transferred onto polyvinylidene fluoride (PVDF) membranes (Millipore, Bedford, MA). The membranes were submersed in 10% skim milk with 0.05% Tween 20 in TBS for overnight at 4 °C and were incubated in mouse anti-His polyclonal antibody (GE Healthcare, Chicaco, IL) at a dilution of 1:3,000 (2 h, room temperature). After washing with TBS-0.05% Tween (TBST), the membrane was incubated with goat anti-mouse IgG-HRP (Sigma Co., St. Louis, MA) at a concentration of 1:5,000 (1 h, room temperature). The reactivity of the antibody was detected by an enhanced chemiluminescence method using an ECL kit (Amersham Biosciences, Buckinghamshire, UK).

### Calcineurin enzymatic activity assay

The recombinant calcineurin enzymatic activity was performed using a colorimetric calcineurin phosphatase activity assay kit (Abcam, Cambridge, MA) based on a Malachite green assay. Briefly, the rHcCNA, rHcCNB, calmodulin and calcineurin assay buffer (100 mM Tris, pH 7.5, 1 mM DTT, 0.05% NP-40, 1 mM CaCl_2_) were mixed, the RII calcineurin substrate (Enz et al., 1994) was added, and the reaction was terminated by adding a Malachite green reagent. After allowing color to develop for 20 min, optical density (OD 620 nm) was read on a Versamax tunable microplate reader (Molecular Devices, San Jose, CA). The absorbance data were converted into released phosphate amount, and calcineurin activity was calculated as a ratio of phosphate amount/reaction time.

### Analysis of calcium-binding activity

The properties of the Ca^2+^-binding activity of the rHcCNB were investigated by ruthenium red staining. The rHcCNB was resolved by SDS-PAGE and was then transferred to a PVDF membrane prior to staining with Ponceau S or ruthenium red (25 mg/l of ruthenium red in 60 mM KCl, 5 mM MgCl_2_, 10 mM Tris–HCl, pH 7.5) for 48 h (Will et al., 2007). Bovine serum albumin (BSA) was used as a negative control.

### The recombinant calcineurin treatment in vivo

To investigate the role of the recombinant calcineurin in shell regeneration, the shell notching experiments were performed according to the method described above. After notching, the abalones were randomly divided into 3 groups, each contained 9 individuals. In all experimental groups, test substances were injected into foot tissue. Group 1 animals (control) were injected with 50 µl of sterile saline solution (0.85% NaCl). Group 2 animals were injected with 100 µg of rHcCNB (about 10 µg/g animal) in 50 µl sterile saline solution. Group 3 animals were injected with 200 µg of rHcCNB (about 20 µg/g animal) in 50 µl sterile saline solution. All animals were maintained in seawater tanks and a subset of abalones from each group were sacrificed after 1, 2 and 3 weeks to collect their shells for measurements of the length and area of regenerated shells and to observe ultrastructure. The length of regenerated shell was measured perpendicular to the shell circumference, from the proximal apex of the original notch to the edge of the new shell growth. The area of regenerated shell represented the area of the original triangular notch that had been infilled with new shell growth (Fig. S1). Regenerated length and area were analyzed by using ImageJ software (http://rsb.info.nih.gov/ij/), and were expressed as a percentage of the original notch length and area to account for minor differences in original notch size.

### Scanning electron microscopy (SEM)

To visualize the growth of 27 regenerated shells, the internal connective tissues of the shell were removed and the shells were washed extensively with distilled water. Thereafter, the shells were left to air-dry in petri dishes at room temperature for several days, cut into 1 cm ×1 cm pieces, and sputter coated with gold using a SPI gold coater (West Chester, PA). The ultrastructural features of the inner surface of the regenerated shells were observed by a Jeol Neoscope JCM-5000 at 15 kV.

### Statistical analysis

Statistical analyses were performed using a one-way ANOVA test followed by a post hoc analysis (Tukey’s multiple comparison test) using IBM SPSS Statistics Processor (IBM, Armonk, NY). Data were expressed as mean ± standard deviation. A *P*-value (*p*) of <0.05 was considered statistically significant.

## Results

### *HcCNA* and *HcCNB* expression after shell notching

**Figure 1:**
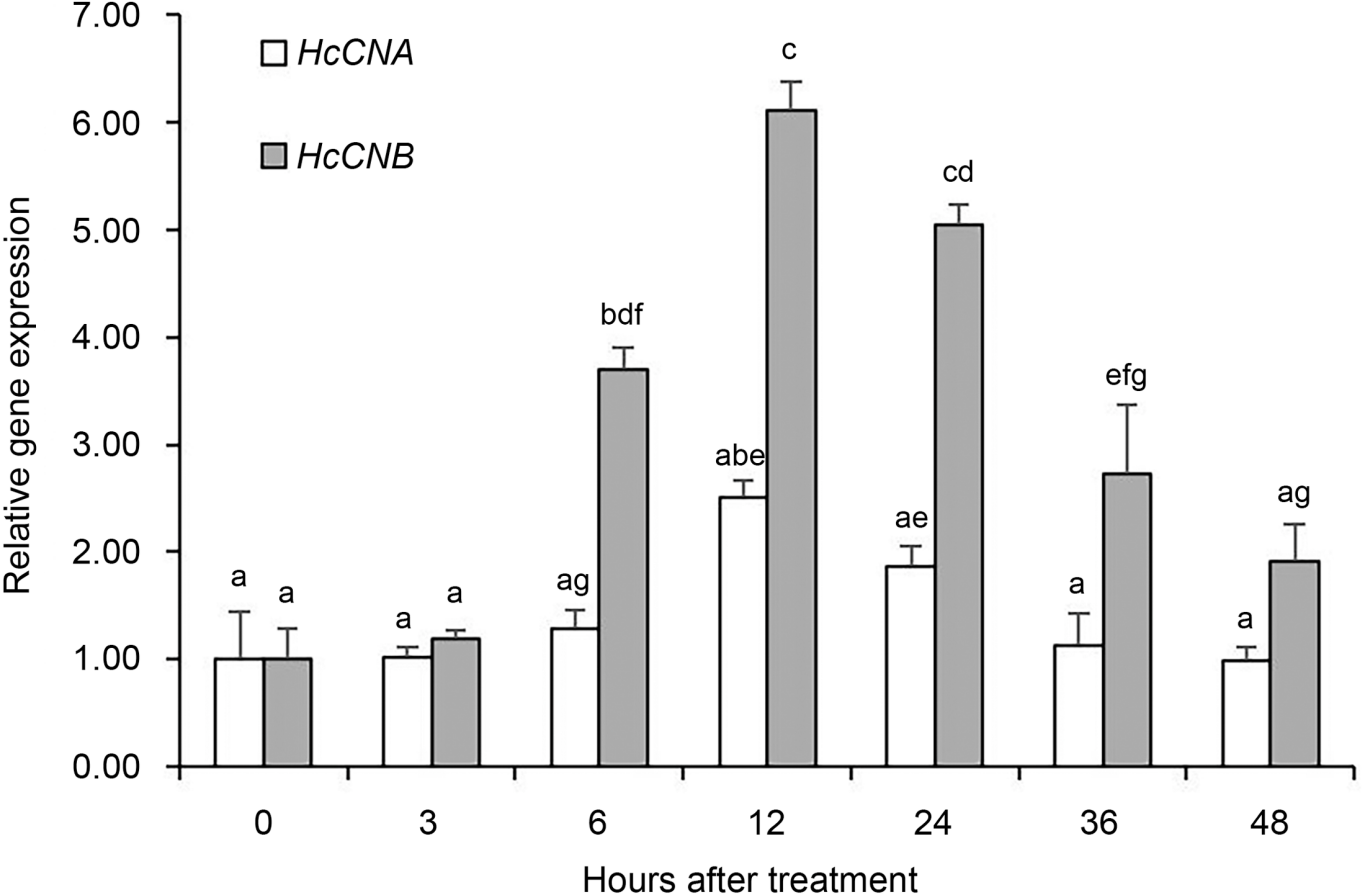
Quantitative real-time PCR analysis of *HcCNA* and *HcCNB* gene expression after shell notching. The relative expression was calculated by the Livak method (2^-ΔΔCq^) using *β*-actin as a reference gene. Data was presented as mean relative expression ±SD. Bars with different letterings indicate a significant difference (*p* < 0.05) while bars with the same letterings indicate non-significant differences.

To monitor the response of *HcCNA* and *HcCNB* genes in the shell regeneration process, the expression levels of *HcCNA* and *HcCNB* genes were examined by quantitative real-time PCR at each time point after shell notching (Fig. 1). All PCR assays displayed efficiency between 91–97% and R^2^ values were more than 0.995. It was noted that the expression level of *HcCNA* was up-regulated at 6 h after shell notching and reached the highest level of about 2.5 fold at 12 h. Thereafter, it was down-regulated at 24, 36, and 48 h. In contrast, the expression level of *HcCNB* significantly increased to almost 4 times higher than *HcCNA* at 6 h and peaked at approximately 3 times that of the *HcCNA* level at 12 h after shell notching. Thereafter, expression of *HcCNB* gradually decreased, however expression levels remained higher than that of the levels of *HcCNA* at the same time intervals.

### Purified recombinant HcCNA and HcCNB proteins in *E. coli* exerted their phosphatase activity

**Figure 2:**
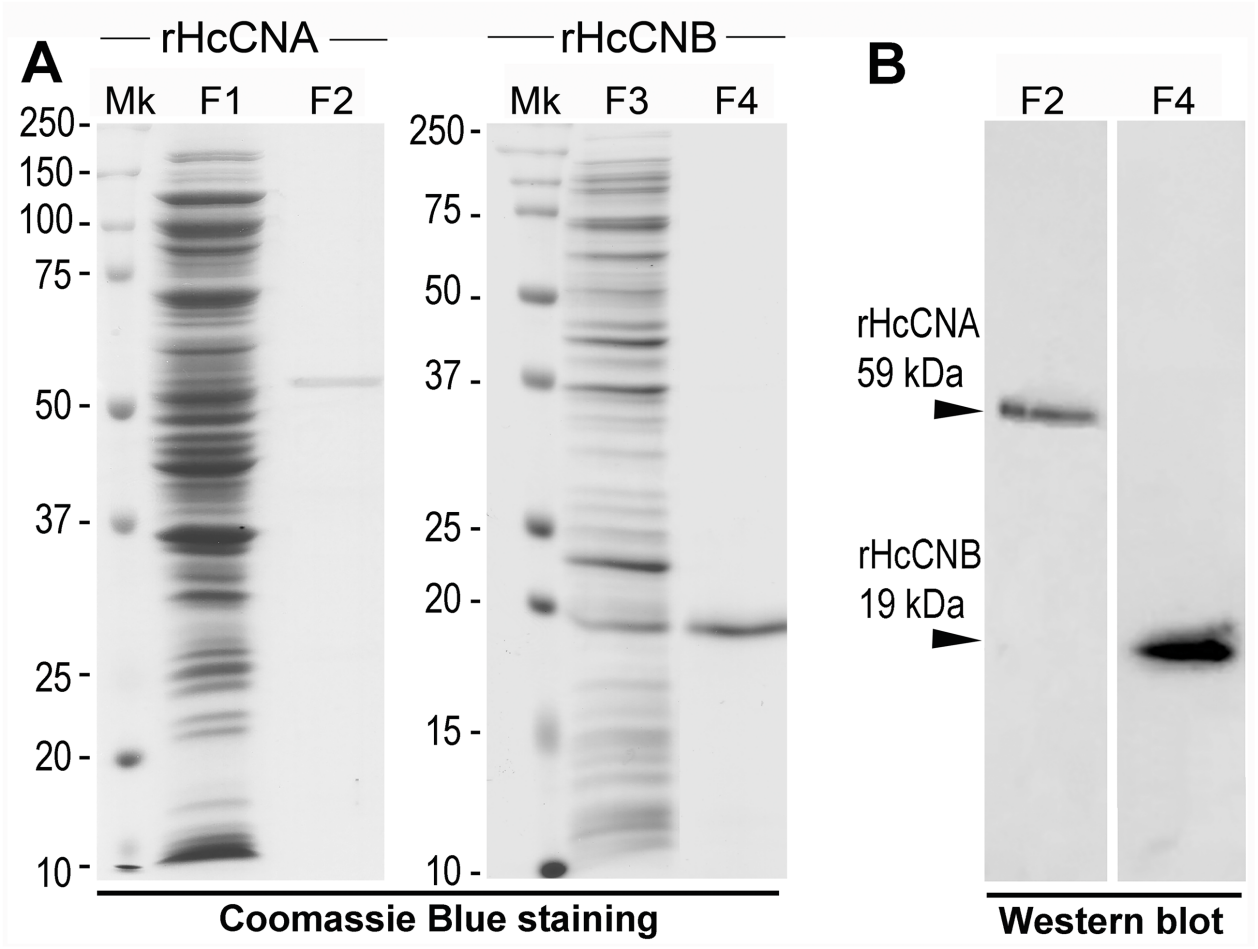
Expression and purification of rHcCNA, rHcCNB and their Western blot analysis. SDS-PAGE gels stained with Coomassie Brilliant Blue showed a single band of purified rHcCNA and rHcCNB (A). (B) represents Western blot analysis of the purified rHcCNA and rHcCNB (arrowheads) using anti-His antibody. Mk, protein molecular mass marker; Lanes F1 and F3, crude lysates; Lanes F2 and 4, purified proteins.

We produced the recombinant proteins, rHcCNA and rHcCNB, in *E. coli* and initially tested the general phosphatase activity as well as the property of Ca^2+^-binding activity of the rHcCNB. Upon its purification with Ni-NTA chromatography, the rHcCNA protein was visible as a single band with an approximate purity of >95%. The protein yield per one purification was approximately 260 µg/mg bacterial extract. Its apparent molecular mass was approximately 59 kDa which was consistent with the predicted molecular mass of HcCNA (Fig. 2A). In a Western blot using an anti-His antibody, only a single immunoreactive band at 59 kDa was observed (Fig. 2B). Similar results were obtained for purification of rHcCNB, where >95% purity and a yield of about 300 µg/mg crude protein was obtained. As also shown in Figs. 2A and 2B, the expressed rHcCNB was shown as a single-band purified protein at about 19 kDa which was consistent with the predicted molecular mass of HcCNB and a single band at 19 kDa was observed by a Western blot analysis with monoclonal anti-His antibody.

**Figure 3:**
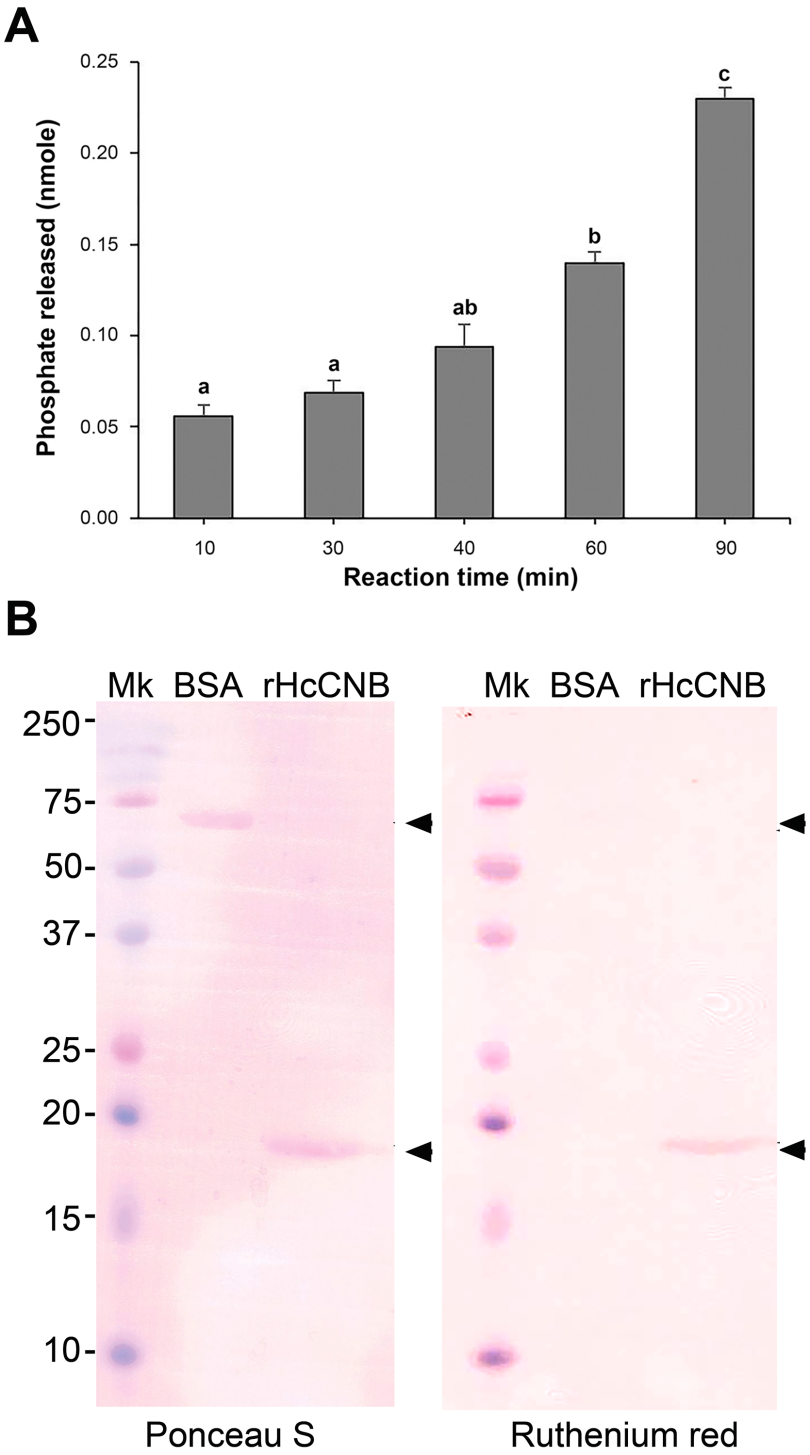
Testing of phosphatase activity of the combined rHcCNA and rHcCNB and Ca^2+^-binding activity of rHcCNB protein. The values of the enzymatic reaction were expressed as nmole phosphate released by minutes of reaction (A). BSA and rHcCNB (arrowheads) were transferred to PVDF membranes and stained with Ponceau S for detection of the proteins (B, Ponceau S) or ruthenium red for detection of Ca^2+^-binding activity (B, Ruthenium red). Mk, protein molecular mass marker.

Generally, the phosphatase function of calcineurin is dependent on dimerization of the calcineurin subunits as well as the presence of a calmodulin catalytic counterpart (Rusnak & Mertz, 2000). In this study, we tested this enzymatic combination followed by the detection with a Malachite green RII calcineurin substrate to measure the amount of phosphate released at different reaction time points. Approximately 0.05 nmol of phosphate was detected at 10 min. This level gradually increased at 30, 40, 60 min and the highest amount of phosphate released was approximately 0.2 nmol at 90 min of the reaction time (Fig. 3A). Together, the results suggested that purified rHcCNA and rHcCNB produced individually from *E. coli* exerted comparable phosphatase activity to that previously reported for CN (Rusnak & Mertz, 2000).

In addition to examination of the phosphatase activity of both subunits, the Ca^2+^-binding activity of rHcCNB was investigated by colorimetric assays using ruthenium red (Fig. 3B). Ruthinium red which has been widely used for analysis of Ca^2+^-binding of proteins, was found to achieve the same results as the ^45^Ca^2+^ overlay technique, and also stained known calcium-binding proteins such as the EF hand Ca^2+^-binding proteins (Charuk, Pirraglia & Reithmeier, 1990; Corbalan-Garcia, Teruel & Gomez-Fernandez, 1992). Here, after the proteins were separated on SDS-PAGE, they were transferred into a PVDF membrane and stained with Ponceau S or ruthenium red. The rHcCNB was stained by ruthenium red, but BSA (the negative control) was not stained, suggesting that rHcCNB had Ca^2+^-binding activity.

### The effect of rHcCNB on the regenerated shell length and area

**Figure 4:**
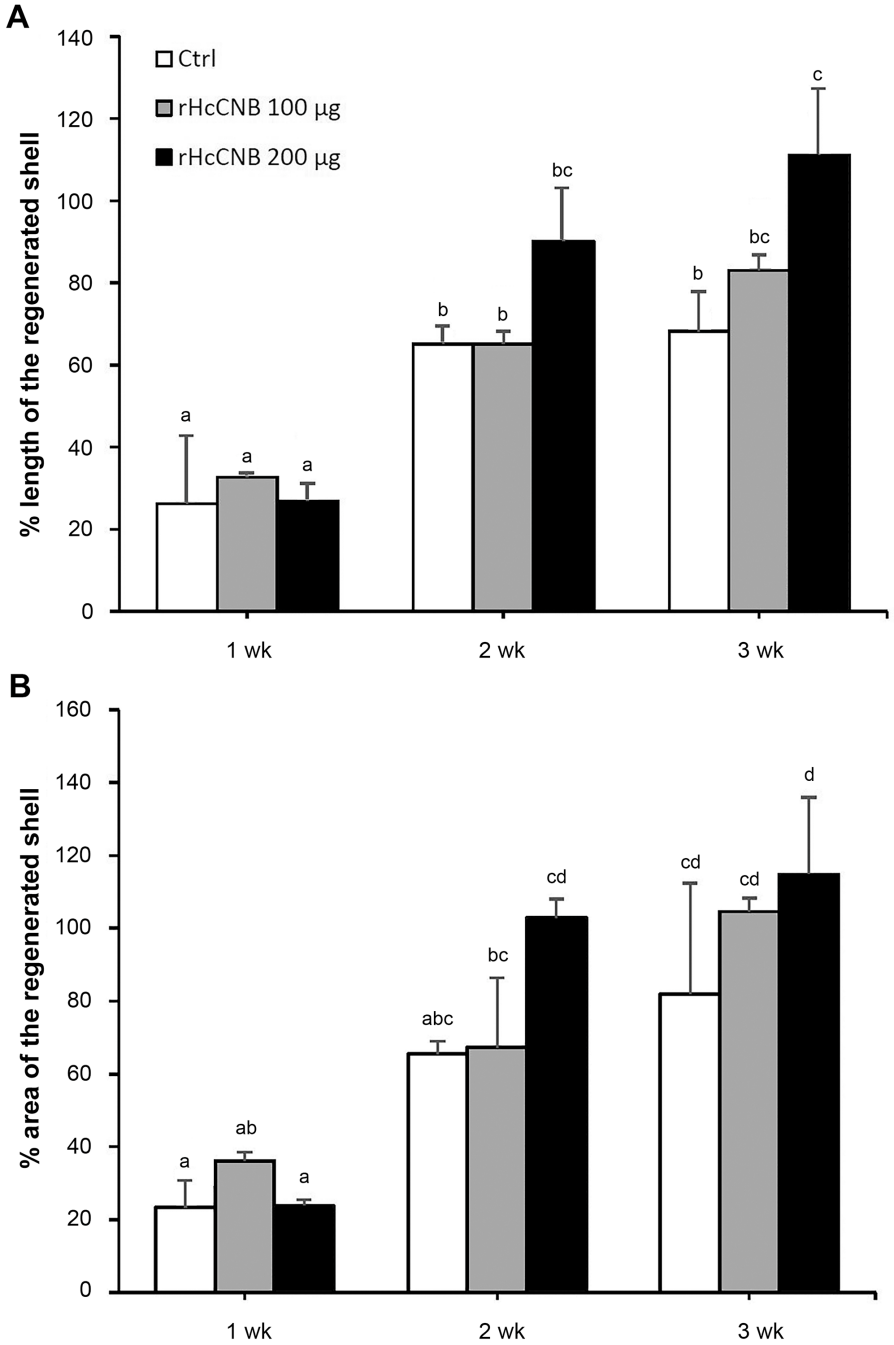
Parameters of the regenerated shells at different periods after shell notching and treatment with rHcCNB. The data of percent length (A) and percent area (B) of shell regenerated portions were calculated from triplicate experiments and expressed as mean ± S.D. Bars with different letterings indicate a significant difference (*p* < 0.05) while bars with the same letterings indicate non-significant differences. Ctrl represents the 0.85% NaCl injected group.

Since our qPCR results indicated that *HcCNB* was significantly up-regulated more than *HcCNA* after shell notching, and that the role of CNB as an independent modulator has been well documented, we decided to further investigate the independent effect of rHcCNB on shell regeneration in vivo. The animals were injected with 0.85% NaCl, 100 µg, or 200 µg of rHcCNB as described above and their shells were collected at 1, 2, and 3 weeks after the treatment. There was no abalone mortality observed and all animals showed evidence of shell regeneration. The regenerated shell length and area were shown as the percentages of the notched region that had been refilled. The percent shell length from three groups were shown in Fig. 4A and their representative images were shown in Figs. S1A–S1C. At one and two weeks after treatment, the percentages of the regenerated shell lengths of all groups showed no significant difference. At three weeks after treatment, the regenerated shell lengths of abalones in Group 3 were significantly higher than that of control groups. The percent shell growth at week 3 (Group 3) exceeded 100% of the original length of the notch. On the other hand, the percentages of regenerated areas showed no significant difference compared to the control at all weeks after treatment (Fig. 4B).

### The effect of rHcCNB on the ultrastructure of the regenerated shell

We further investigated ultrastructure of the regenerated shell upon treatment with rHcCNB. Scanning electron microscopy of the inner surface of the regenerated shell revealed three distinct zones (Fig. S1D). The outermost, or prismatic (P), layer was located at the edge of the regenerated shell. The innermost layer, the nacreous (N) layer, was located proximally to the prismatic layer. A band with distinct ultrastructure that was located between the prismatic and nacreous layers was named transition zone (T).

**Figure 5:**
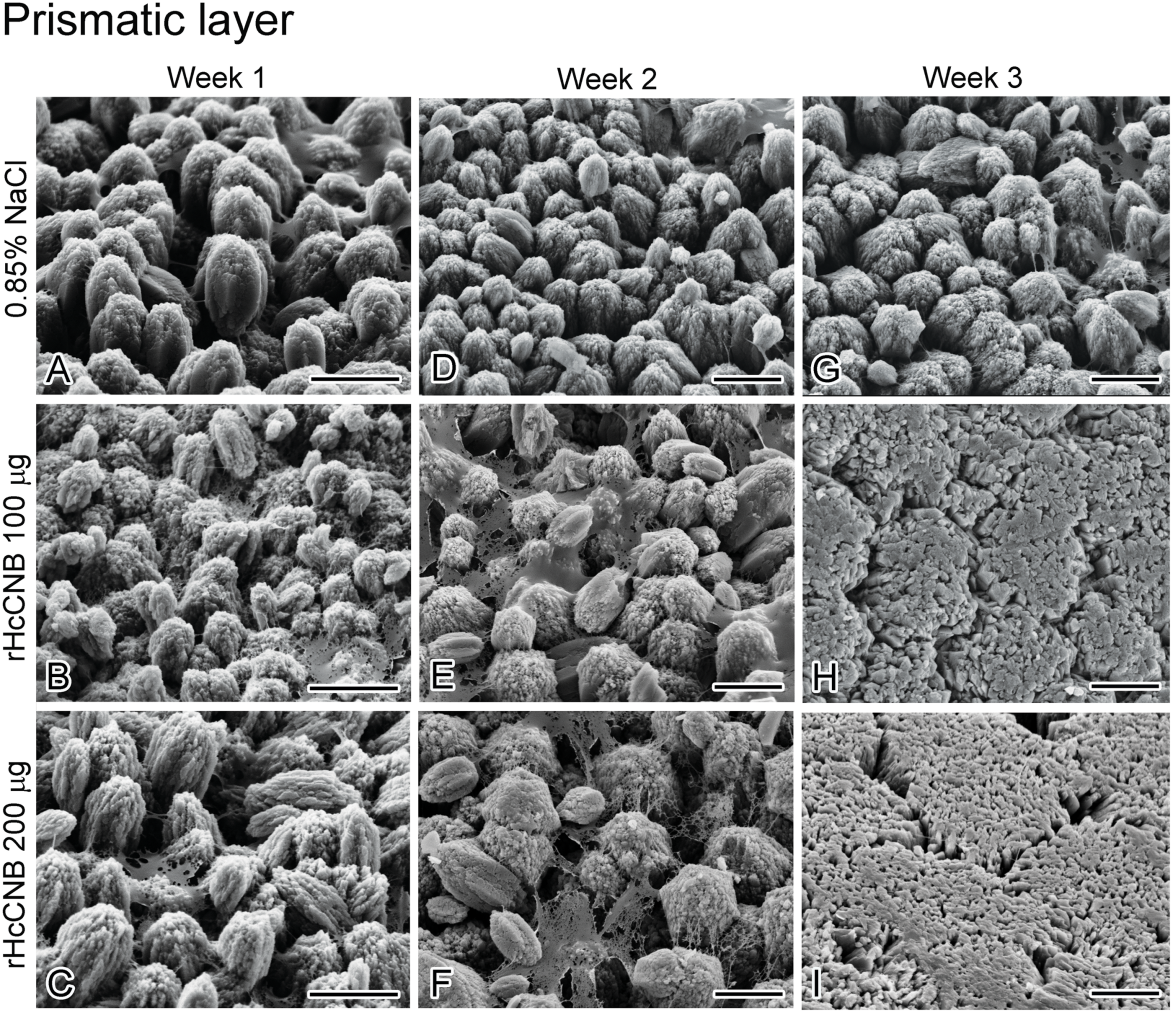
The regenerated prismatic layer at the shell’s inner surface after shell notching and rHcCNB treatment. SEM images of week 1, 2, and 3 after shell notching and rHcCNB treatment were shown in the left, middle, and right panels, respectively. The prismatic layers of the animals injected with 100 µg of rHcCNB were shown in B, E, and H and those injected with 200 µg of rHcCNB were shown in C, F, and I. The controls (injection of 0.85% NaCl) were shown in A, D, and G. Bars = 5 µm.

**Figure 6:**
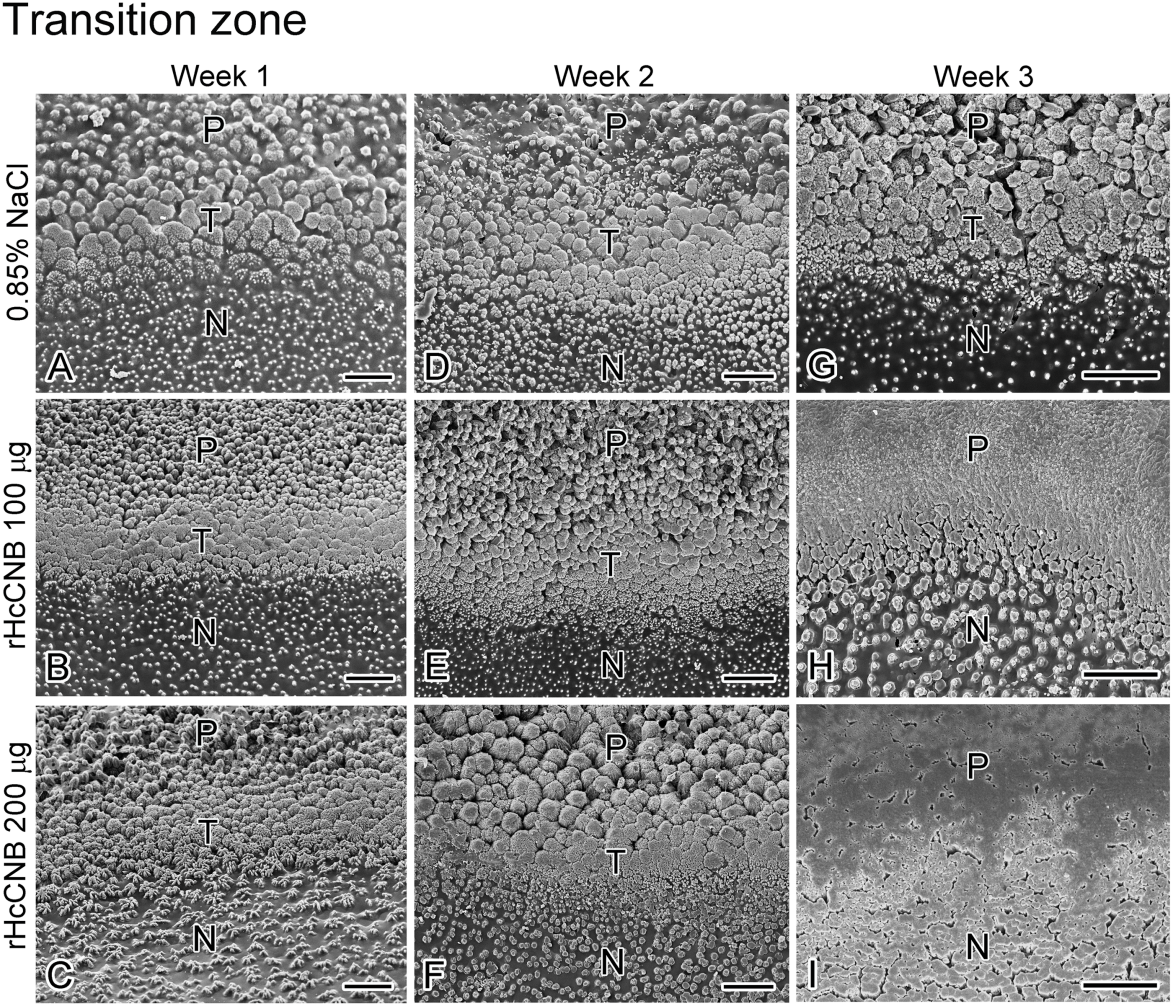
The transition zone (T) at the shell’s inner surface after shell notching and rHcCNB treatment. SEM images of week 1, 2, and 3 after shell notching and rHcCNB treatment were shown in the left, middle, and right panels, respectively. The transition zone of the animals injected with 100 µg of rHcCNB were shown in B, E, and H and those injected with 200 µg of rHcCNB were shown in C, F, and I. The controls (injection of 0.85% NaCl) were shown in A, D, and G. P, prismatic layer; N, nacreous layer; T, transition zone; Bars = 25 µm.

**Figure 7:**
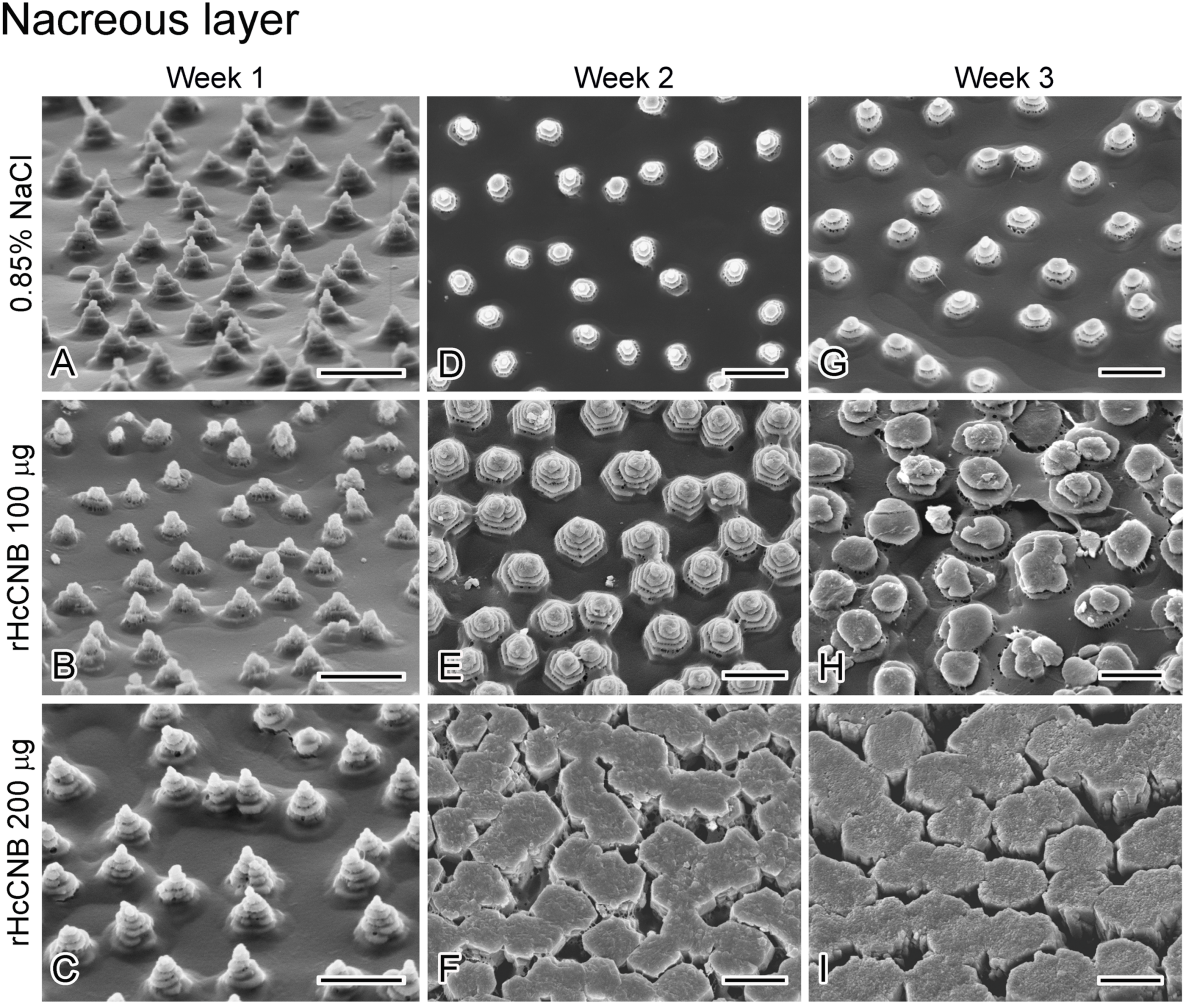
The regenerated nacreous layer at the shell’s inner surface after shell notching and rHcCNB treatment. SEM images of week 1, 2, and 3 after shell notching and rHcCNB treatment were shown in the left, middle, and right panels, respectively. The nacreous layers of the animals injected with 100 µg of rHcCNB were shown in B, E, and H and those injected with 200 µg of rHcCNB were shown in C, F, and I. The controls (injection of 0.85% NaCl) were shown in A, D, and G. Bars = 5 µm.

Upon treatment with rHcCNB after shell notching, the inner surfaces of the regenerated shells changed their structures drastically compared with the control group. At weeks 1 and 2 after shell notching and treatment, the ultrastructure of prismatic layers of all groups contained numerous small fine-grained crystals that aggregated to form various sized prolate spheroids (Figs. 5A–5F). At week 3, the ultrastructure of the crystals differed significantly between control (Fig. 5G) and rHcCNB injected animals (Figs. 5H–5I). The structure at the transition zone (T) at weeks 1 and 2 was not different from the control group (Figs. 6A–6F). The transition zones were easily identifiable, containing fine-grained crystals that aggregated to form larger lattices. Of particular interest, the transition zone was not observed in the rHcCNB injected animals at week 3 after treatment (Figs. 6H–6I). In the nacreous layer, the polygonal crystal tablets of regenerated shell generally resembled the pyramidal stacks of the control group at week 1 (Figs. 7A–7C). However at week 2 and 3, the organization of tablets did not appear as stacks in Group 3, but rather as discontinuous brick columns. The diameter of the columns in Group 3 was much larger than that of the stacked tablets, resulting in the reduction of the space between units (Figs. 7F and 7I).

## Discussion

Regeneration or repair of the fractured shell is dependent mostly on secretory contents from the mantle epithelium (Marin et al., 2008) and partly from hemocytes (Mount et al., 2004; Kadar, Tschuschke & Checa, 2008). The epithelial cells of the mantle secrete matrix proteins, transport various ions through ion channels, and form a supersaturated environment in the extrapallial space for shell biomineralization (Mann, 2001). The outer epithelium of the mantle that is located adjacent to the fractured shell is known to be important for this process (Fleury et al., 2008). In some species of marine invertebrates, the microstructures observed in regenerated shell differs from the normal shell (Tsujii, 1976). It is interesting to note that different areas of shell damage may elicit different types of shell regeneration processes or mechanisms. Shell repair after drilling a hole approximately 1 cm away from the shell edge in *H. tuberculata* does not recapitulate normal shell mineralogy, but produces a stratified mineral organization consisting of up to six microstructures in the newly grown material (Fleury et al., 2008). In contrast, shell notching at the shell edge (as studied herein) resulted in three different zones with unique ultrastructures in the regenerated areas (Figs. 5–7). This type of mineral arrangement may exhibit a superimposition of different microstructures which differs from the mineral organization pattern found in normally developing shells of many mollusks (Marin, Roy & Marie, 2012). This discrepancy of mineral organization between normal shells and repaired shells reflects the variable function of the mantle epithelium in the two different situations.

Among the many proteins that are known to be involved in shell biomineralization (Zhang & Zhang, 2006), we have shown herein that HcCNB plays a role in shell mineralization and regeneration processes in *H. diversicolor.* Firstly, *HcCNB* was up-regulated to a greater extent than *HcCNA* within the mantle upon shell notching (Fig. 1). Secondly, administration of rHcCNB significantly enhanced the growth of regenerated shell at the notched areas (Figs. 4–7). Both subunits of calcineurins (CN) have been known to act synergistically to elicit phosphatase activity via the formation of heterodimeric complex between the two subunits (Rusnak & Mertz, 2000). To regulate phosphatase activity, the regulatory CNB is required to interact irreversibly with the N-terminal CNB-binding domain of CNA. The CNA-CNB complex then binds either to Ca^2+^ ions directly (EF-hand motif of CNB) or to calmodulin-Ca^2+^ (on CNA) to activate the phosphatase pocket on CNA (Thewes, 2018). Recently, evidence has been accumulated to support the independent function of CNB without forming a heterodimeric complex with CNA. Recombinant CNB protein in isolation has been reported to prevent pellet aggregation in a similar manner as Ca^2+^ chelators or other antiplatelet aggregation substances (Su et al., 2011). As CNB contains four EF-hand motifs (Rusnak & Mertz, 2000), its Ca^2+^ chelating function could thus be expected. In fact, the independent function of CNB resembles that of calmodulin, a protein that is also in the family of EF-hand Ca^2+^-binding proteins and is involved in regulation of Ca^2+^ uptake, transport, and secretion, in shell formation of the pearl oyster *P. fucata* (Li et al., 2004; Yan et al., 2007). Interestingly, the EF-hand Ca^2+^-binding domain-containing novel *P. fucata* protein, EFCBP (which contains just 2 EF-hand motifs) has also been shown to participate in shell regeneration by responding quickly to shell damage (Huang et al., 2007). Together, these results suggest that EF-hand Ca^2+^-binding domain-containing proteins have important roles in shell repair in bivalves and gastropods.

Despite a significant up-regulation of *HcCNB* within 6–48 h of shell notching, differences in mineral accumulation (shell morphology) between treated and control shells were not notable in the initial stages of shell repair. Three weeks after rHcCNB treatment, however, the morphology of the regenerated shells were clearly different compared to controls, particularly in the prismatic layer. The crystal organization no longer resembled fine-grained crystal units, but rather denser lattice-like structures, in the two rHcCNB groups (Figs. 5H and 5I). In the 3-week treatment period we also noticed an increase in size of nacre tablets, a morphological change from a tablet stack to a discontinuous brick column (Figs. 7G and 7I), and the absence of transition zone (Figs. 6H and 6I). In fact, analysis of the ultrastructure of regenerating shells after rHcCNB treatment has raised two areas for further investigation. Firstly, it is apparent that rHcCNB increased the extent of shell regeneration, and therefore the apparent change in mineral organization may be the consequence of CNB-induced accelerated mineralization. One possible mechanism is via increased recruitment of Ca^2+^ into the site of mineralization, mediated by the Ca^2+^-binding ability of the EF-hand motifs found within CNB, however this remains to be tested. Secondly, the latent morphological effect of rHcCNB delivery indicates that the mechanism by which it acts on biomineral formation may require additional, as yet unknown, spatial and/or temporal factors. It is also worth noting that two dimensional (2D) morphometric analysis of the regenerated shell (both length and area) may not provide the best parameters to differentiate the change in shell growth. Here we demonstrated a significant change in the percentage of regenerated shell length between treatments and controls, but not in the percentage of regenerated shell area. This may be due to the geometric organization of shell materials in three dimensions —the thickness increases gradually towards the center of the shell. Such shell geometry would undoubtedly introduce a complication for morphometric analysis which should be considered using volumetric (3D) analysis. The combination of both 2D and 3D analyses as well as comparative weight measurements of the regenerated shell should be considered to improve geometrical material analysis.

In conclusion, both calcineurin genes of *H. diversicolor* (but predominantly *HcCNB*) were up-regulated after shell notching. Injection of rHcCNB protein accelerated shell repair and caused altered shell ultrastructure after three weeks. These results demonstrate that HcCNB can function to influence shell regeneration in gastropod mollusks.

##  Supplemental Information

10.7717/peerj.10662/supp-1 Supplemental Information 1 qPCR of HcCNA and HcCNB. Raw data of qPCR and analysis of quantification cycle values (Cq) using the Livak method (2^−ΔΔ^ Cq) at different time points (0, 3, 6, 12, 24, 36, 48 h) after shell notching.

10.7717/peerj.10662/supp-2 Supplemental Information 2 Western blot of rHcCNA and rHcCNB. In panel A and B, arrowheads indicated positive bands of rHcCNA at 59 kDa and rHcCNB at 19 kDa in** crude of *E. coli* lysate and Elute 1 of protein purification.

10.7717/peerj.10662/supp-3 Supplemental Information 3 Calcineurin enzymatic activity. Raw data of calcineurin enzymatic activity assay indicated the absorbance data (OD) that were converted into phosphate released (PO4).

10.7717/peerj.10662/supp-4 Supplemental Information 4 Measurement of shell length and area. Raw data of the original notching parameters and analyzation the growth of shell in terms of shell length and area.

10.7717/peerj.10662/supp-5 Supplemental Information 5 Stereomicrographs and low magnification SEM of shell notching areas after treating with rHcCNB or 0.85% NaCl for 1, 2, and 3 weeks The black and white dot lines indicate borders of shell growth in week 1- 3 (A, B, C). SEM image (D) of the inner shell surface from the boxing area of B indicates the 3 layers of shell regeneration. P, prismatic; T, transition zone; N, nacreous; Bars in A–C = one mm; Bar in *D* = 200 µm.

## Additional Information and Declarations

### Data Availability

The following information was supplied regarding data availability: Raw data, including qPCR data, uncropped western blot analysis, enzymatic activity data, changes in length and area of shell growth, and stereomicrograph and low magnification SEM of shell notching areas, are available in the Supplemental Files.
