# Supplementary figures and images for: Calcineurin subunit B is involved in shell regeneration in Haliotis diversicolor
Source: PeerJ. 2021 Jan 12;9:e10662. doi: 10.7717/peerj.10662 (PMC7810044; doi:10.7717/peerj.10662)

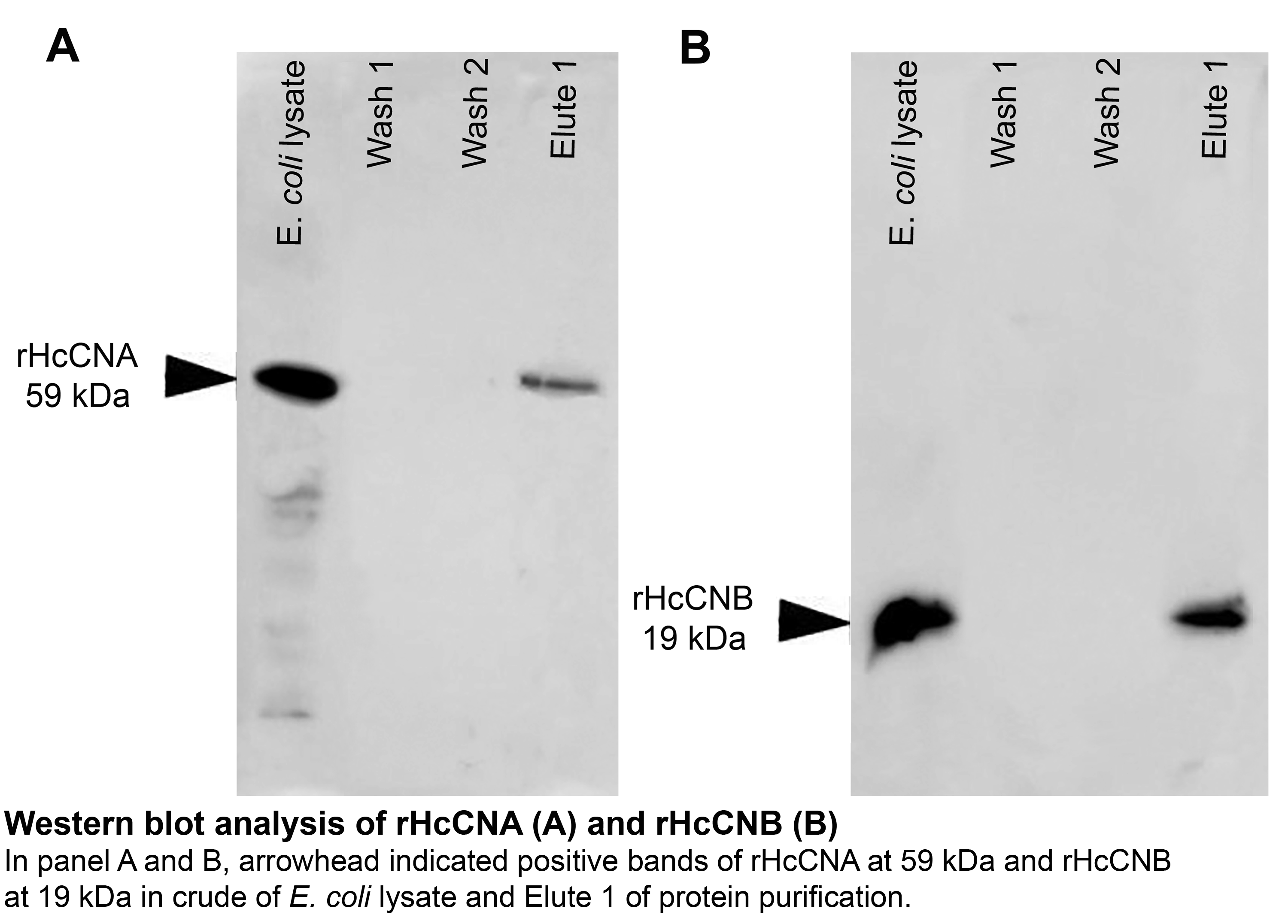

Supplement: Supplemental Information 2 — In panel A and B, arrowheads indicated positive bands of rHcCNA at 59 kDa and rHcCNB at 19 kDa in crude of E. coli lysate and Elute 1 of protein purification. [file peerj-09-10662-s002.png]

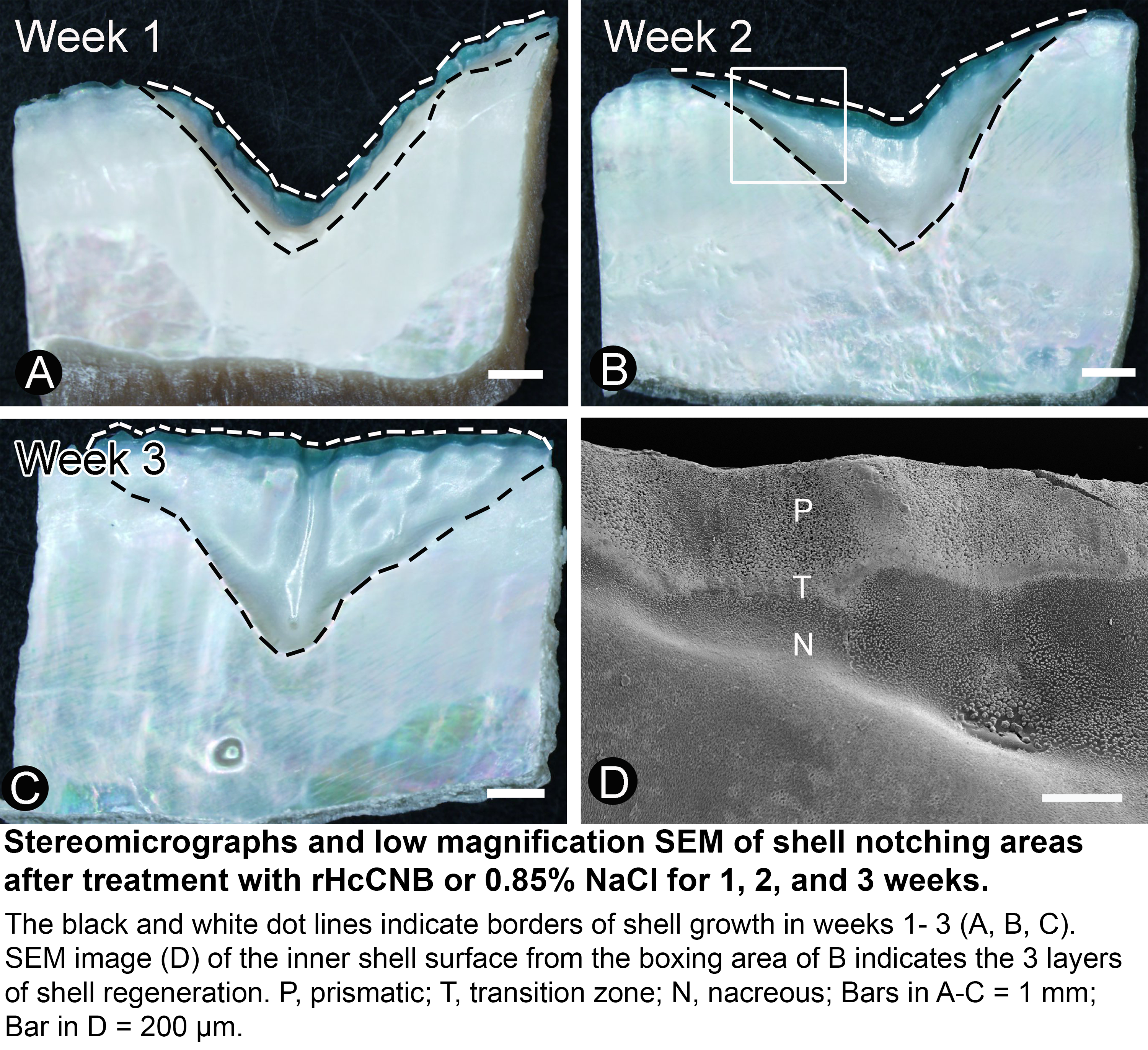

Supplement: Supplemental Information 5 — The black and white dot lines indicate borders of shell growth in week 1- 3 (A, B, C). SEM image (D) of the inner shell surface from the boxing area of B indicates the 3 layers of shell regeneration. P, prismatic; T, transition zone; N, nacreous; Bars in A–C = one mm; Bar in D = 200 µm. [file peerj-09-10662-s005.png]
